# Hospital admissions for non-communicable disease in the UK military and associations with alcohol use and mental health: a data linkage study

**DOI:** 10.1186/s12889-020-09300-5

**Published:** 2020-09-10

**Authors:** L. Goodwin, D. Leightley, Z. E. Chui, S. Landau, P. McCrone, R. D. Hayes, M. Jones, S. Wessely, N. T. Fear

**Affiliations:** 1 Department of Psychology, University of Liverpool, Room 2.31 Eleanor Rathbone Building, Liverpool, L69 7ZA UK; 2 King’s Centre for Military Health Research, Institute of Psychiatry, Psychology & Neuroscience, King’s College London, London, UK; 3 Liverpool Centre for Alcohol Research, Liverpool Health Partners, Liverpool, UK; 4 Biostatistics & Health Informatics, Institute of Psychiatry, Psychology & Neuroscience, King’s College London, London, UK; 5 Institute for Lifecourse Development, University of Greenwich, London, UK; 6 Psychological Medicine, Institute of Psychiatry, Psychology & Neuroscience, King’s College London, London, UK; 7 Academic Department of Military Mental Health, Institute of Psychiatry, Psychology & Neuroscience, King’s College London, London, UK

**Keywords:** Military, Armed forces, Cohort study, Data linkage, Electronic healthcare records, Non-communicable disease, Alcohol use, Mental health, Post-traumatic stress disorder

## Abstract

**Background:**

Since the recent conflicts in Iraq and Afghanistan, the short-term focus of military healthcare research has been on the consequences of deployment for mental health and on those wounded or injured in combat. Now that these conflicts have ended for the UK Armed Forces, it is important to consider the longer term physical and mental health consequences, and just as importantly, the links between these. The aims of this study were to determine the most common physical conditions requiring a hospital admission in UK military personnel and whether they were more common in personnel with a mental health condition, smokers, and/or those misusing alcohol compared to those without.

**Methods:**

Data linkage of a prospective UK military cohort study to electronic admitted patient care records for England, Wales and Scotland. Nine thousand nine hundred ninety military personnel completed phase 2 of a military cohort study (56% response rate, data collected from 2007 to 2009), with analyses restricted to 86% of whom provided consent for linkage to healthcare records (*n* = 8602). Ninety percent were male and the mean age at phase 2 was 36 years. The outcome was physical non communicable diseases (NCDs) requiring a hospital admission which occurred after phase 2 of the cohort when the mental health, smoking and alcohol use exposure variables had been assessed until the end of March 2014.

**Results:**

The most common NCDs requiring a hospital admission were gastrointestinal disorders 5.62% (95% Confidence Intervals (CI) 5.04, 6.19) and joint disorders 5.60% (95% CI 5.02, 6.18). Number of NCDs requiring a hospital admission was significantly higher in those with a common mental disorder (Hazard ratio (HR) 1.40 (95% CI 1.16–1.68), post-traumatic stress disorder (HR 1.78 (95% CI 1.32–2.40)) and in current smokers (HR 1.35 (95% CI 1.12–1.64) compared to those without the disorder, and non-smokers, respectively.

**Conclusions:**

Military personnel with a mental health problem are more likely to have an inpatient hospital admission for NCDs compared to those without, evidencing the clear links between physical and mental health in this population.

## Background

Since the recent conflicts in Iraq and Afghanistan, the short-term focus of military healthcare research for the Coalition Forces who participated has been on the consequences of deployment for mental health [1, 2] and on those wounded or injured in combat [3]. Now that these conflicts have ended for the UK Armed Forces (AF), it is important to consider the longer term physical and mental health consequences, and just as importantly, the links between these [4].

Comparisons of the physical health of serving and ex-serving personnel to the general population have provided contrasting findings. The most recent data from the U.S. Veterans Eligibility Trends and Statistics showed that life expectancy is shorter by approximately 1 year for ex-serving personnel compared to the general U.S. population, and additionally found the same socioeconomic patterning as in the general population, with higher mortality rates in ex-serving personnel with a lower educational attainment and lower household income [5]. This is supported by evidence that ex-serving personnel report more chronic conditions compared to non-veterans [6] and have an increased risk of coronary heart disease [7], attributed to historically higher rates of smoking [8]. Musculoskeletal disorders and arthritis are also more common in ex-serving personnel than in civilians [9].

Conversely, a healthy warrior or solider effect has been proposed to result from the screening and selection processes that occurs at enlistment. A meta-analysis quantified this theory, with an overall protective effect for all-cause mortality ranging from 10 to 25%, in ex-serving personnel compared to the general population [10] but reported no difference in the UK studies [11, 12]. However, the healthy warrior effect is likely to reduce over time, particularly if there are exposures through military service associated with longer term worsening of physical health.

It is crucial that we take a nuanced approach when examining the type of physical health conditions reported, given that increased all-cause mortality in ex-serving personnel may be explained by specific causes [13]. The current study will focus on hospital admissions for non-communicable diseases (NCDs), which account for approximately two thirds of deaths globally, most commonly due to cardiovascular diseases [14] and provide much needed evidence on the links between mental health and physical NCDs in the UK AF. Much of the data on the physical health of ex-serving personnel has been recorded through US Veterans Affairs (VA) healthcare services [15] (most likely because visits are recorded for payment purposes), or from studies which use self-reports of physical health [16]. VA reports suggest that the most common conditions are hypertension, followed by arthritis, diabetes and ischaemic heart disease [17]. Combat personnel appear to have an additional health burden compared to non-combat exposed personnel, for example with an increased risk of chronic pain and stroke [18] and increased levels of inflammatory markers in trauma exposed Israeli combat ex-serving personnel [19].

There are a number of reasons why the risk of NCDs could be high in serving and ex-serving personnel. First, there is the consequence of serving in a physical demanding occupation (e.g. for musculoskeletal conditions [20]), in addition to the specific health impacts of deploying to challenging environments [21]. Second, there may be predisposing risk factors, relating to higher levels of recruitment to the AF from areas of greater deprivation, associated with a greater risk of childhood adversity [22]. Third, alcohol is consistently identified to be a problem in military populations [23, 24], with an established health burden [25], but an increased smoking prevalence is not consistent across countries [26, 27]. Whilst levels of exercise are found to be higher in serving personnel, they decrease after leaving service [26] with generally poor cardiovascular health metrics in ex-serving personnel [28]. Finally, the mental health of serving personnel appears to be worse than civilians [29], with known physical health consequences.

In keeping with a vast civilian literature, the physical health of ex-serving personnel with a mental health problem is poorer; personnel with post-traumatic stress disorder (PTSD) are more likely to have musculoskeletal, neurological, and gastrointestinal disorders compared to those without PTSD [30]. Behavioural and biological mechanisms have been evidenced to explain the links between PTSD and cardiovascular health, with a strong role of health behaviour risks, such as alcohol and smoking [31]. Depression is linked to an increased risk of mortality in general population studies [32] and specifically to musculoskeletal disorders in veterans, with proposed explanatory factors including chronic pain and physical inactivity [33–35]. Of significance to the current study is the finding that US ex-serving personnel with a mental disorder have a higher number of all cause hospitalisations [36].

The current study focuses on which NCDs (requiring inpatient hospital care) are most common in a UK military population. It will use secondary healthcare records from England, Wales and Scotland, linked to the King’s Centre for Military Health Research (KCMHR) military cohort [1, 37] to identify the healthcare records of a large representative sample of military personnel. It is also the first study of which we are aware to analyse secondary datasets from England, Wales and Scotland together [38]. This paper reports on: 1) the most common NCDs requiring a hospital admission in the UK military; 2) how i) self-reports of alcohol misuse, smoking and mental health problems (reported before the outcomes) and ii) change in alcohol use and mental health are associated with NCDs requiring an admission after adjustment for sociodemographic factors, military characteristics and childhood adversity. Additional analyses will 3) examine the adjusted association between alcohol misuse, smoking and mental health with the total number of NCDs requiring an admission and 4) will conduct age standardised comparisons to publicly available data on NCDs requiring a hospital admission in England in the general population.

## Methods

### Study design

Data linkage was conducted between a large UK military cohort study and electronic secondary healthcare records for admitted patient care in England, Wales and Scotland [38]. The exposures were measured at phases 1 [37] and 2 [1] of the cohort study (described below) and outcomes were reported in the healthcare records from the phase 2 questionnaire completion date onwards until the end of March 2014 (See Supplementary figure 1).

### Data

#### King’s Centre for Military Health Research cohort study

The KCMHR cohort is a large representative study of military personnel. Data were collected in 2004–2006 (phase 1) and again in 2007–2009 (phase 2). Phase 1 recruited approximately 10% of UK military personnel who had been deployed to the first phase of the Iraq war, and a further sample who had not been deployed to Iraq. Ten thousand two hundred seventy-two participants in total responded (8686 Regulars, 1586 Reservists; 59% response rate) [37]. For phase 2 data collection (2007–2009), 9395 participants from phase 1 were available for follow-up. Six thousand four hundred twenty-nine completed the phase 2 data collection (68% response rate). Response at phase 2 was associated with being older, female, an officer and a regular (and so these factors were included in the development of the survey response weights). There were two additional samples at phase 2; with 896 personnel recruited who had deployed to Afghanistan (response rate 50%) and 2665 individuals responding who had joined the military between April 2003 and April 2007 (response rate 40%). In total, 9990 individuals completed the phase 2 questionnaire (overall response rate 56%) [1]. Individuals who took part at phase 1 only are not included in the analyses and 86% of the phase 2 participants provided consent for linkage to healthcare records (*n* = 8602).

#### Socio-demographic, pre-military, military and health characteristics from the KCMHR cohort

##### Socio-demographic variables

Demographic information available was sex, age (at time of phase 2 questionnaire) and marital status at phase 2 (categorised as single or in a relationship/married or separated/divorced/widowed).

##### Military characteristics

Self-reported at phase 2 were military rank (Other ranks/non-commissioned Officer or Officer rank), status (regular or reservist), service (Naval Services or Army or Royal Air Force), serving status (Serving or ex-serving at phase 2), role in parent unit (combat, combat service support or combat support) and most recent deployment (including data on last deployment to Iraq or Afghanistan (or both)) were used.

##### Pre-military characteristics (childhood adversity)

A measure of family relationship childhood adversity was assessed [39] (either at phase 1 or 2 dependent on when personnel joined the cohort), adapted from the Adverse Childhood Exposure study scale [40]. This included 8 items which were summed to form a cumulative measure and analysed as 0/1, 2/3 and 4+ adversities.

##### Smoking

Smoking status and frequency were assessed through a self-report at phase 2 categorised as ex-smoker, non-smoker and current smoker.

##### Mental health and alcohol use at phases 1 & 2

The General Health Questionnaire (GHQ-12) was used to screen for symptoms of common mental disorder (CMD), providing a general assessment of current psychological distress [41, 42]. Examples of items include ‘feeling unhappy or depressed’ and ‘feeling constantly under strain’. For this study the bi-modal scoring method of 0–0–1-1 was used, with those endorsing a negative symptom as ‘rather’ or ‘much more than usual’, or a positive symptom as ‘less’ or ‘much less than usual’, classified as reporting a symptom. Scores for the full scale ranged from 0 to 12 and a > =4 cut-off was used to represent probable CMD. Symptoms of PTSD (in line with the Diagnostic and Statistical Manual of mental disorders (DSM-IV) [43]) were assessed by the National Centre for PTSD Checklist – Civilian version (PCL-C [44];); a 17-item questionnaire assessing five re-experiencing, seven avoidance and five hyperarousal symptoms, which has previously been used in military populations [1]. The PCL-C was used in preference to the military version because it is less restrictive in considering traumatic events unrelated to deployment. Cases were defined as individuals with a total score of 50 or above, referred to as ‘probable PTSD’. Alcohol use was measured by the 10-item World Health Organization (WHO) Alcohol Use Disorders Identification Test [45]. A score of 0–7 was classified as low risk drinking, 8–15 indicated hazardous use, a score of 16 or more was used to define harmful alcohol misuse (hazardous use, likely to be harmful to health) and individuals who reported currently never drinking were categorised separately (when cell sizes where large enough, otherwise this category was combined with low risk drinking). A single item from the AUDIT was used to characterise binge drinking “How often do you have six or more drinks on one occasion?”, with those endorsing 6 or more units weekly or more defined as a binge drinker. The main analyses only included these variables assessed at phase 2 as the exposure variables. Variables reflecting change in mental health/alcohol status from phase 1 to 2 were derived for CMD and alcohol misuse (but not for PTSD due to low numbers). These all included 4 categories: no case stable, case stable (at both phases), positive change (from case to no case) and negative change (no case to case).

### Hospital admissions in electronic healthcare records (EHRs)

Secondary care visits for physical health conditions for regulars, reservists and veterans take place either through Ministry of Defence Hospital Units that are hosted within National Health Service (NHS) hospitals or directly through NHS hospitals (including visits commissioned by the Ministry of Defence). This study combined three NHS datasets from NHS Digital (for Hospital Episode Statistics; HES), Information Services Division (ISD) and Secured Anonymised Information Linkage (SAIL) which cover all NHS secondary care in England, Scotland and Wales, respectively. The EHR data were requested for the financial years 2003/04 to 2013/2014 (in order to cover the timescale of the KCMHR cohort study with some follow-up after phase 2). The outcomes assessed in the current study were restricted to those occurring in admitted patient care (APC) episodes as outpatient and accident & emergency data did not have acceptable completion of formalised diagnosis codes [38]. For the survival analyses, an event was considered as occurring from the time of the phase 2 questionnaire onwards and individuals with the event between phases 1 and 2 were excluded.

### Data linkage

#### Linkage of the administrative dataset for the cohort participants to EHRs

A dataset of unique patient identifiers including NHS number, forename, surname, sex and date of birth, and a unique scrambled cohort identifier to allow for linkage back to the cohort, was provided to each of the three Nations [38]. Linkage was conducted separately by NHS Digital for England, NHS Information Services for Wales and ISD for Scotland, using different matching procedures (see [46, 38] for additional details on the linkages).

#### Integrating the EHRs

Records and episodes relating to APC events for the separate HES, SAIL and ISD datasets were combined for variables which were co recorded across all three Nations, including primary and secondary diagnoses coded to International Classification of Diseases, 10th revision (ICD-10, [47, 48]). The combined EHR data from across the Nations was then merged to the KCMHR cohort data using the scrambled cohort identifier.

### Data access and cleaning methods

A number of data checks were conducted, including checking admission and discharge dates for chronological consistency. Diagnoses for each participant were reviewed and the first chronological occurrence of a diagnosis was identified and these data were stored in a date format.

### Bias

Efforts were taken to reduce potential sources of bias by comparing any differences between the matched and unmatched samples. This suggested that the greatest difference between these samples was by presence of NHS number, which would be expected given this was used in the matching process. The final analyses used the full consented sample (*n* = 8602) as the denominator for analyses, rather than only those who were matched (*n* = 6336). This takes a potentially conservative approach in reporting prevalence estimates and associations, rather than over inflating estimates if the matched sample were more likely to have a secondary care event (personnel with an NHS number were both more likely to be matched and may also have been more likely to have visited an NHS organisation). However, the key analyses reported in Table 4 were repeated as sensitivity analyses in the matched only sample and the differences in the results compared to analysing the full sample were very minimal (results reported in Supplementary table 2).

### Health outcomes in the electronic healthcare records

Diagnoses across England, Wales and Scotland were coded using the three-character (ICD-10, [47]) categories which either group diagnoses that share common characteristics or represent single conditions [49]. The health outcomes of interest in the current study were physical non-communicable diseases (NCDs), identified in the healthcare records using the relevant ICD-10 codes. There is no generic framework of NCDs recommended for electronic healthcare records, so a systematic review was conducted to identify which NCDs codes to extract (ICD-10). This review resulted in a framework of 28 non-communicable physical conditions (which excluded infectious disease, mental health disorders and conditions relating to reproduction and fertility) under ten disease categories (reported in Supplementary file 1).

### Data analysis

This study included the full sample of those who consented to allow access to their medical records (*n* = 8602). Combined sampling weights accounted for both the over-sampling of particular groups (e.g. reservists) at phase 1 and the probability weights of non-response at phase 2. All frequencies were unweighted and weighted proportions and weighted model estimates were reported using the survey commands in STATA v.14 [50]. A complete case analysis approach was used. Missing data on the exposure and confounding variables from the KCMHR cohort data (which all 8602 participants completed) ranged from 0 to 1.1%. Cell sizes less than 8 were not reported according to NHS Digital guidelines [51].

1. **Frequencies and weighted % were calculated for the full consented sample (*n*** **= 8602) and the matched (*n*** **= 6336) and unmatched samples (*n*** **= 2266)** for all socio-demographics, military characteristics, childhood adversity, and smoking, alcohol and mental health variables (Supplementary table 1). Weighted Chi square (χ^2^) analyses were conducted comparing the matched sample with those who were not matched. The Pearson χ2 statistic with the Rao and Scott [52] second-order correction was presented (Supplementary table 1).
2. **Weighted proportions and 95% confidence intervals were calculated for all 28 NCDs requiring a hospital admission from the new framework**, **with the top 10 presented ordered by proportion.** These were first calculated for any events which occurred across the full study period, between 2003/04 to 2013/14, and were then calculated for events occurring after the date of phase 2 questionnaire completion. The sample included all participants who had consented to their hospital records to be matched, minus those individuals with the event before the date they completed the phase 2 questionnaire (Table 1).
3. **The top 5 NCDs were cross tabulated with all of the sociodemographic, military characteristics and childhood adversity, and– unweighted frequencies, weighted % and confidence intervals are reported.** Unadjusted weighted Cox regression analyses identified where there were statistically significant associations with these potential confounding variables and the NCD outcomes, restricted to NCD events that occurred after the completion of the phase 2 questionnaire and using the date of the admission as the outcome. The median number of total NCDs (out of the 28 NCDs studied) was calculated overall and then selecting individuals separately with each of the top 5 NCDs (Table 2).
4. **Age standardised comparisons with the general population for England** – For the top 5 conditions, frequency data from HES (England only) were compared to publicly available HES data for the combined ICD-10 code categories (stratified by age). Age standardised proportions in the military and general population data were calculated and then used to calculate numbers per 1000 for both populations (these data were restricted to men only for prostate disorders). Publicly available HES data was not available stratified by both gender and age together, which is why only the age standardised data were presented.
5. **Weighted negative binomial regression models were conducted with the number of NCD conditions occurring after phase 2 as the outcome (*n*** **= 8518).** Number of NCDs was treated as a non-normally distributed count variable and reflected the number of NCDs requiring an inpatient admission after phase 2, with mental health, alcohol use and smoking status the exposure variables in the models. Participants with an admission for any NCD before phase 2 and with no admissions after phase 2 were excluded from this analysis, as the analyses aimed to predict new NCD events after phase 2. The models were tested in a number of steps, i) unadjusted models, ii) adjusted for age, gender, marital status and military characteristics (rank, serving status, engagement type, service branch, deployment to Iraq or Afghanistan and primary role in parent unit), iii) additionally adjusted for family relationship adversity in childhood, and iv) the models with probable PTSD and CMD as the key exposures were additionally adjusted for alcohol misuse and smoking status at phase 2.
6. **Weighted cox proportional hazards models were conducted for each of the top 5 NCD outcomes –** The outcome for the models was the date for the first event for each NCD that occurred after the completion of the phase 2 questionnaire and the regressions examined mental health, alcohol use and smoking status as the exposures in the models. The adjustments were the same as for the negative binomial regression models in 5) with adjustments i)–iv). Any participants with the NCD event before phase 2 were excluded from the analyses. Checks for proportional hazards assumptions were tested using Schoenfeld residuals. If the global χ^2^ test was statistically significant, this indicated that the proportional hazards assumption had been violated. All of the global χ^2^ tests were found to be non-significant.
7. **Weighted Cox regression models were conducted restricted to participants who completed both phases 1 and 2 of the cohort (*n*** **= 5794) to examine change in CMD status across phase 1 and 2 as the exposure (numbers were too low to run change models for probable PTSD and alcohol misuse).** Change was categorised as not a case stable (did not meet criteria at phase 1 or 2), case stable (met criteria both phases), positive change (met criteria at phase 1 but not at phase 2) and negative change (did not meet criteria at phase 1 but did by phase 2). The adjustments were the same as for the previous models in 2) and 6).

**Table 1:** The top 10 NCDs in the full sample (*n* = 8602) ordered by % (overall and after completion of phase 2 questionnaire)

**Rank**	**Events overall (*n* = 8602)**	**n**	**Weighted % (95% CI)**	**New events after phase 2 (n differs by condition)**^**a**^	**n**	**Weighted % (95% CI)**
**1**	**Gastrointestinal Disorders**	482	5.62 (5.04, 6.19)	**Gastrointestinal Disorders**	303/8423	3.64 (3.17, 4.12)
**2**	**Other Joint Disorders**	467	5.60 (5.02, 6.18)	**Other Joint Disorders**	291/8426	3.38 (2.93, 3.83)
**3**	**Arthritis/osteoarthritis**	183	2.18 (1.82, 2.55)	**Arthritis/osteoarthritis**	131/8550	1.54 (1.23, 1.85)
**4**	**Hypertension**	151	2.02 (1.66, 2.37)	**Hypertension**	105/8556	1.38 (1.09, 1.68)
**5**	**Prostate and other genitourinary disorder**	158	1.94 (1.59, 2.29)	**Prostate and other genitourinary disorder**	90/8534	1.14 (0.87, 1.41)
**6**	**Asthma/COPD**	130	1.59 (1.27, 1.91)	**Asthma/COPD**	88/8560	1.01 (0.76, 1.26)
**7**	**Back and neck pain**	123	1.56 (1.25, 1.88)	**Back and neck pain**	76/8555	0.92 (0.68, 1.16)
**8**	**Cancer and Tumours**	99	1.30 (1.01, 1.59)	**Cancer and Tumours**	67/8570	0.89 (0.65, 1.14)
**9**	**Migraine/headache**	88	1.00 (0.75, 1.25)	**Hyperlipidaemia**	55/8589	0.72 (0.51, 0.94)
**10**	**Hyperlipidaemia**	68	0.90 (0.66, 1.14)	**Migraine/headache**	53/8567	0.68 (0.47, 0.89)

^a^Participants were excluded who had an APC event for that condition before phase 2 so the numbers reflect the numbers included in the later Cox regression analyses

**Table 2:** Top 5 NCDs by demographic, military characteristics and childhood adversity

	**Full sample characteristics (*n* = 8602)**	**Gastrointestinal Disorders (*n*** **= 303)**	**Other Joint Disorders (*n*** **= 291)**	**Arthritis/Osteoarthritis (*n*** **= 131)**	**Hypertension (*n*** **= 105)**	**Prostate and other genitourinary diseases (*n*** **= 90)**
**Age in years at phase 2 (Mean (S.D.)) (n = 8602)**	36.02 (9.20)	38.85 (9.42)***	35.70 (9.00)	41.29 (8.61)***	44.63 (9.41)***	38.30 (9.34)
	**n (weighted proportion, 95% CIs)**	**n (weighted proportion with condition, 95% CIs)**	**n (weighted proportion with condition, 95% CIs)**	**n (weighted proportion with condition, 95% CIs)**	**n (weighted proportion with condition, 95% CIs)**	**n (weighted proportion with condition, 95% CIs)**
**Sex (n = 8602)**						
Male	7661 (90.49; 89.8–91.1)	267 (3.65; 3.15–4.16)	269 (3.50; 3.02–3.99)	118 (1.55; 1.23–1.88)	(*)	90 (1.26; 0.96–1.56)
Female	941 (9.50; 8.80–10.1)	36 (3.51; 2.11–4.91)	22 (2.12; 1.03–3.22)	13 (1.35; 0.46–2.25)	(*)	0
**Relationship status at phase 2 (*n*** **= 8575)**						
In a relationship	6602 (79.07; 78.06–80.05)	244 (3.80; 3.29–4.39)	228 (3.42; 2.95–3.98)	112 (1.66; 1.34–2.06)	86 (1.49; 1.18–1.88)	77 (1.24; 0.96–1.60)
Not in a relationship	1973 (20.92; 19.95–21.94)	59 (3.10; 2.26–4.23)	63 (3.24; 2.41–4.33)	19 (1.11; 0.66–1.87)	19 (1.02; 0.59–1.76)	13 (0.78; 0.41–1.49)
**Rank at phase 2 (*n* = 8602)**						
Officer	1930 (20.03; 19.08–20.98)	86 (4.26; 3.21–5.32)	60 (2.99; 2.11–3.87)	38 (1.76; 1.11–2.41)	26 (1.57; 0.90–2.23)	20 (0.95; 0.45–1.45)
NCO and other ranks	6672 (79.96; 79.01–80.91)	217 (3.48; 2.95–4.02)	231 (3.47; 2.95–3.99)	93 (1.48; 1.13–1.8)	79 (1.33; 1.00–1.67)	70 (1.18; 0.87–1.50)
**Left service marker (*n*** **= 8588)**						
Serving	6471 (71.34; 70.18–72.50)	208 (3.17; 2.65–3.68)	236 (3.63; 3.09–4.17)	93 (1.43; 1.08–1.77)	57 (0.94; 0.66–1.22)	62 (1.04; 0.73–1.34)
Discharged	2117 (28.65; 27.49–29.81)	95 (4.84; 3.77–5.91) **	55 (2.74; 1.92–3.57)	38 (1.81; 1.16–2.45)	48 (2.50; 1.72–3.27)***	28 (1.38; 0.81–1.96)
**Regular/Reserve status (*n* = 8602)**						
Regular	7059 (88.72; 88.07–89.38)	225 (3.46; 2.95–3.97)	235 (3.36; 2.87–3.85)	105 (1.52; 1.19–1.85)	77 (1.2; 0.95–1.58)	76 (1.14; 0.85–1.44)
Reserve	1543 (11.27; 10.61–11.92)	78 (5.07; 3.75–6.39)*	56 (3.47; 2.40–4.55)	26 (1.67; 0.91–2.44)	28 (2.29; 1.34–3.24)*	14 (1.10; 0.44–1.76)
**Service (*n* = 8602)**						
Naval Services	1317 (16.38; 15.45–17.31)	32 (2.87; 1.78–3.97)	47 (3.70; 2.52–4.89)	25 (1.79; 0.97–2.61)	18 (1.53; 0.76–2.31)	10 (0.73; 0.2–1.2)
Army	5628 (64.16; 62.9–65.36)	228 (4.20; 3.56–4.8)	207 (3.66; 3.08–4.24)	85 (1.51; 1.13–1.88)	65 (1.30; 0.94–1.66)	69 (1.35; 0.9–1.71)
RAF	1657 (19.44; 18.45–20.43)	43 (2.46; 1.60–3.33)**	37 (2.15; 1.34–2.97)**	21 (1.40; 0.72–2.09)	22 (1.50; 0.79–2.22)	11 (0.79; 0.24–1.3)
**Deployed to Iraq or Afghanistan before phase 2 (*n* = 8602)**						
No deployment	2809 (43.55; 42.30–44.81)	118 (4.35; 3.52–5.19)	84 (3.00; 2.30–3.70)	(*)	49 (1.95; 1.39–2.52)	32 (1.27; 0.81–1.74)
Iraq only	3696 (37.19; 36.0–38.37)	127 (3.29; 2.59–3.99)	133 (3.53; 2.82–4.24)	(*)	37 (0.93; 0.56–1.30)**	33 (0.91; 0.54–1.29)
Afghanistan only	917 (8.821; 8.153–9.489)	27 (2.83; 1.50–4.16)	27 (3.29; 1.8–4.76)	(*)	8 (1.21; 0.26–2.15)	11 (1.04; 0.27–1.80)
Iraq and Afghanistan	1180 (10.42; 9.733–11.11)	31 (2.64; 1.49–3.79)	47 (4.41; 2.94–5.87)*	(*)	11 (0.76; 0.22–1.29)*	14 (1.46; 0.53–2.38)
**Role in parent unit at phase 2 (*n*** **= 8512)**						
Combat	2035 (24.01; 22.94–25.08)	52 (2.79; 1.91–3.68)	78 (3.76; 2.79–4.73)	27 (1.40; 0.78–2.01)	19 (1.05; 0.51–1.58)	16 (0.81; 0.34–1.28)
Combat support	1011 (11.41; 10.63–12.19)	38 (4.25; 2.69–5.81)	34 (3.20; 1.93–4.48)	12 (1.20; 0.39–2.01)	10 (1.32; 0.43–2.20)	12 (1.33; 0.48–2.10)
Combat service support	5466 (64.57; 63.37–65.76)	209 (3.84; 3.23–4.44)	178 (3.30; 2.74–3.86)	91 (1.64; 1.25–2.03)	75 (1.53; 1.13–1.92)	62 (1.24; 0.88–1.60)
**Family relationship adversities in childhood (*n*** **= 8510)**						
0/1	5479 (64.22; 63.00–65.41)	195 (3.67; 3.11–4.31)	179 (3.26; 2.78–3.86)	88 (1.64; 1.29–2.09)	56 (1.15; 0.86–1.54)	55 (1.06; 0.78–1.44)
2/3	1720 (19.94; 18.96–20.96)	53 (3.10; 2.23–4.23)	60 (3.28; 2.41–4.45)	22 (1.14; 0.68–1.90)	24 (1.58; 0.99–2.51)	13 (0.83; 0.46–1.52)
4+	1311 (15.84; 14.94–16.79)	52 (4.30; 3.12–5.90)	52 (4.14; 3.02–5.65)	21 (1.71; 1.03–2.82)	25 (2.18; 1.40–3.37)*	22 (1.93; 1.19–3.09)*
**Number of NCDs (median, IQR) (*n* = 8602)**	0 (0–0)	1 (1–2)	1 (1–2)	2 (2–3)	1 (1–3)	2 (2–4)

Statistically significant association with the NCD outcome in unadjusted Cox regression analysis (restricted to events occurring after phase 2) at *p < 0.05, **p < 0.01, ***p < 0.001

(*) – Numbers and percentages are not reported when n is smaller than 8 due to Hospital Episode Statistics reporting guidelines

## Results

### Overview of the data linkage

The overall number of participants from the cohort matched to electronic healthcare records from either England, Wales or Scotland was 6336 (73%). Participants who were matched were more likely to have a NHS number (Supplementary table 1), with only a small number matched without an NHS number in Wales and Scotland due to differences in the approaches utilised. The matching process for England required an NHS number. Of the total number of individuals matched, 4460 were matched in England only (71%), 257 were matched in Wales only (4%), 826 in Scotland only (13%) and 793 were matched in more than one region (12%). Younger personnel, personnel in the Army, who reported a previous deployment to Iraq and those in a combat role in their parent unit were more likely to be matched. Those who reported childhood antisocial behaviour, who met the criteria for harmful alcohol use on the AUDIT and who self-reported a CMD were also more likely to be matched (Supplementary table 1), probably because they were more likely to have used NHS services.

### Overview of the sample

The full sample (Table 2 and Supplementary Table 1) were on average aged 36 years at the time of the phase 2 questionnaire and could have been up to approximately 8 years older by the time of hospital admission. Ninety percent were male and 78% married or in a relationship. Twenty percent were of Commissioned Officer rank and 28% had left the services by phase 2. Fifty six percent had previously deployed to Iraq or Afghanistan, or to both, and 24% held a combat role in their parent unit. Thirty five percent reported 2 or more family relationship adversities in childhood. Forty three percent of the sample met AUDIT criteria for hazardous drinking and 13% met criteria for harmful drinking. Binge drinking was very common, with 38% binge drinking more than weekly. Twenty four percent were current smokers. Four percent met criteria for probable PTSD and 20% of the sample for a CMD.

### Healthcare use (admitted patient care)

Thirty nine percent of the analysed sample had an APC event. As expected, the majority of APC events were at English hospitals, with 36, 4 and 5% of the sample having APC events at English, Welsh and Scottish hospitals, respectively.

### Top 10 non-communicable diseases

The median number of NCDs requiring a hospital admission in the full sample was zero. The most common NCDs requiring an admission in the full sample were gastrointestinal (GI) disorders and joint disorders, both over 5% (Table 1). The next frequent were arthritis/osteoarthritis (2%), prostate and genitourinary (GU) disease (2%) and hypertension (2%). Cancer and tumours was the eighth most common (1%). When restricted to admissions which occurred after completion of the phase 2 questionnaire, the proportions were lower but the order stayed the same, other than for migraine/headaches and hyperlipidaemia for which the order reversed.

GI disorders were more common in older personnel, those who had left services, reserves, personnel who had served for 12 or more years and less likely in personnel in the Royal Air Force (Table 2). Joint disorders were less common in personnel in the RAF and more common in those who had deployed to both Iraq & Afghanistan. Arthritis and osteoarthritis were more common in older personnel and those who had served for more than 12 years. Prostate and GU disorders were less common in personnel who were single and with four or more family relationship adversities in childhood. Hypertension was more common in older personnel, those who had left service, reserves, those with longer military service, and in those who reported four or more family relationship adversities in childhood. Hypertension was less common in those who had deployed to Iraq or to both Iraq and Afghanistan.

### Age standardised comparisons with the general population (per 1000 individuals)

Comparisons to HES data from the general population (standardised by age) showed that GI disorders (15.2 (military) vs 41.9 (gen pop) per 1000) and hypertension (22.8 vs 40.1) appeared to be more common in the general population, but joint problems (23.5 vs 7.4) and arthritis (19.9 vs 13.1), and prostate disorders (18.1 vs 7.4, in males only) were more common in the military population (full details of these calculations available from the authors).

### Associations with number of NCDs reported

Number of NCDs requiring a hospital admission was significantly associated with both CMD and probable PTSD, with a stronger association with probable PTSD. Current smokers reported a greater number of NCDs (Table 3).

**Table 3:** Negative binomial regression models showing the association between mental health, alcohol misuse and smoking with number of NCDs (*n* = 8452)

	**Median number of NCDs (IQR)**^**a**^	**Unadjusted Incidence Rate Ratio (IRR) (95% CI)**	**Adjusted IRR (95% CI)**^**b**^	**Adjusted IRR (95% CI)**^**c**^	**Adjusted IRR (95% CI)**^**d**^
**PTSD**					
Non-case	1 (1–2)	1.00	1.00	1.00	1.00
Case	1 (1–2)	**1.86 (1.39–2.48)**	**1.91 (1.42–2.56)**	**1.78 (1.34–2.37)**	**1.78 (1.32–2.40)**
**CMD**					
Non-case	1 (1–2)	1.00	1.00	1.00	1.00
Case	1 (1–2)	**1.35 (1.12–1.61)**	**1.41 (1.19–1.69)**	**1.38 (1.16–1.65)**	**1.40 (1.16–1.68)**
**Alcohol misuse**					
Current never drinkers	2 (1–2)	1.13 (0.67–1.92)	1.22 (0.68–2.20)	1.21 (0.67–2.18)	
Low risk drinking (0–7)	1 (1–2)	1.00	1.00	1.00	
Hazardous drinking (8–15)	1 (1–2)	0.87 (0.73–1.04)	1.00 (0.84–1.19)	1.00 (0.84–1.18)	
Harmful drinking/possible dependence (16+)	1 (1–1)	**0.76 (0.60–0.97)**	1.03 (0.80–1.31)	1.02 (0.80–1.31)	
**Binge drinking at phase 2**					
Monthly or less	1 (1–2)	1.00	1.00	1.00	
Weekly or more	1 (1–2)	**0.84 (0.71–0.99)**	0.95 (0.81–1.11)	0.96 (0.81–1.12)	
**Smoking status at phase 2**					
Non-smoker	1 (1–2)	1.00	1.00	1.00	
Ex-smoker	1 (1–2)	**1.28 (1.07–1.52)**	1.22 (0.94–1.34)	1.11 (0.94–1.33)	
Smoker	1 (1–2)	**1.34 (1.09–1.65)**	**1.36 (1.12–1.65)**	**1.35 (1.12–1.64)**	

^a^Weighted medians and interquartile ranges are reported for those who had at least one NCD

^b^Adjusted for age, gender, marital status, rank, serving status, engagement type, service branch, deployment to Iraq or Afghanistan and primary role in parent unit

^c^Additionally adjusted for family relationship adversity in childhood

^d^Additionally adjusted for alcohol misuse and smoking status

### Alcohol, smoking and mental health as predictors of the top 5 NCDs

#### Gastrointestinal disorders

Individuals with probable PTSD and/or CMD had 2 and 1.5 times the hazard, respectively, of having an admission for a GI disorder in the fully adjusted model, compared to those without the mental disorder (Table 4). Smokers had 1.5 times the hazard of an admission for a GI disorder compared to non-smokers, after all adjustments. The analyses restricted to participants who completed both phases 1 and 2 examined change in CMD status across the same time period, as exposures for NCD admissions occurring after phase 2 (Table 5). Personnel with CMD at both phases, those who became a CMD case by phase 2 (i.e. did not meet criteria at phase 1, but did by phase 2) and those who met CMD criteria at phase 1 only, had 2, 1.8 and 1.7 times the hazard, respectively, of having an admission for a GI disorder after phase 2, in the fully adjusted models.

**Table 4:** Cox proportional hazards models examining the associations with mental health, alcohol misuse, smoking for the top 5 NCDs

	**n with disorder (row %)**	**Unadjusted Hazard Ratio (HR) (95% CI)**	**Adjusted HR (95% CI)**^**a**^	**Adjusted HR (95% CI)**^**b**^	**Adjusted HR (95% CI)**^**c**^
**Gastrointestinal disorders (*n*** **= 8359, failures = 303)**					
**PTSD**					
Non-case	279 (3.46%)	1.00	1.00	1.00	1.00
Case	21 (7.58%)	**2.29 (1.38–3.81)**	**2.15 (1.27–3.63)**	**2.11 (1.24–3.61)**	**2.28 (1.34–3.89)**
**CMD**					
Non-case	230 (3.35%)	1.00	1.00	1.00	1.00
Case	72 (4.85%)	**1.47 (1.07–2.02)**	**1.48 (1.08–2.2)**	**1.49 (1.08–2.03)**	**1.55 (1.12–2.15)**
**Alcohol misuse**					
Low risk drinking (0–7)^d^	138 (3.92%)	1.00	1.00	1.00	
Hazardous drinking (8–15)	130 (3.51%)	0.92 (0.69–1.22)	1.04 (0.76–1.41)	1.03 (0.76–1.41)	
Harmful drinking/possible dependence (16+)	30 (3.27%)	0.89 (0.56–1.41)	1.10 (0.68–1.77)	1.07 (0.65–1.77)	
**Binge drinking at phase 2**					
Monthly or less	201 (4.17%)	1.00	1.00	1.00	
Weekly or more	97 (2.85%)	**0.70 (0.52–0.93)**	0.76 (0.56–1.03)	0.76 (0.56–1.03)	
**Smoking status at phase 2**					
Non-smoker	134 (2.96%)	1.00	1.00	1.00	
Ex-smoker	85 (3.97%)	1.32 (0.96–1.82)	1.18 (0.85–1.64)	1.17 (0.84–1.63)	
Smoker	77 (4.45%)	**1.56 (1.12–2.16)**	**1.65 (1.19–2.28)**	**1.64 (1.18–2.28)**	
**Joint disorders (*n*** **= 8361, failures = 291)**					
**PTSD**					
Non-case	272 (3.34%)	1.00	1.00	1.00	1.00
Case	15 (4.04%)	1.23 (0.67–2.26)	1.23 (0.66–2.27)	1.18 (0.64–2.17)	1.07 (0.57–1.99)
**CMD**					
Non-case	224 (3.31%)	1.00	1.00	1.00	1.00
Case	62 (3.59%)	1.08 (0.77–1.52)	1.06 (0.76–1.49)	1.05 (0.74–1.47)	1.00 (0.70–1.43)
**Alcohol misuse**					
Low risk drinking (0–7) ^d^	117 (3.18%)	1.00	1.00	1.00	
Hazardous drinking (8–15)	129 (3.26%)	1.04 (0.77–1.39)	0.98 (0.72–1.33)	0.97 (0.71–1.32)	
Harmful drinking/possible dependence (16+)	42 (4.55%)	1.47 (0.98–2.22)	1.33 (0.86–2.05)	1.32 (0.85–2.06)	
**Binge drinking at phase 2**					
Monthly or less	169 (3.09%)	1.00	1.00	1.00	
Weekly or more	118 (3.86%)	1.27 (0.96–1.67)	1.21 (0.91–1.61)	1.21 (0.91–1.61)	
**Smoking status at phase 2**					
Non-smoker	124 (2.80%)	1.00	1.00	1.00	
Ex-smoker	78 (3.52%)	1.25 (0.89–1.74)	1.25 (0.89–1.75)	1.24 (0.88–1.74)	
Smoker	85 (4.43%)	**1.63 (1.17–2.25)**	**1.54 (1.10–2.16)**	**1.53 (1.09–2.15)**	
**Arthritis/osteoarthritis (*n*** **= 8484, failures = 131)**					
**PTSD**					
Non-case	120 (1.49%)	1.00	1.00	1.00	1.00
Case	8 (2.22%)	1.55 (0.68–3.52)	1.76 (0.77–4.02)	1.78 (0.79–4.01)	1.73 (0.71–4.20)
**CMD**					
Non-case	98 (1.52%)	1.00	1.00	1.00	1.00
Case	31 (1.65%)	1.10 (0.68–1.78)	1.19 (0.74–1.91)	1.20 (0.75–1.94)	1.10 (0.66–1.85)
**Alcohol misuse**					
Low risk drinking (0–7) ^d^	62 (1.80%)	1.00	1.00	1.00	
Hazardous drinking (8–15)	55 (1.34%)	0.78 (0.51–1.19)	0.91 (0.58–1.42)	0.92 (0.59–1.44)	
Harmful drinking/possible dependence (16+)	10 (1.16%)	0.70 (0.33–1.49)	0.95 (0.44–2.09)	0.99 (0.44–2.20)	
**Binge drinking at phase 2**					
Monthly or less	83 (1.62%)	1.00	1.00	1.00	
Weekly or more	44 (1.35%)	0.86 (0.56–1.33)	0.98 (0.63–1.53)	0.99 (0.64–1.55)	
**Smoking status at phase 2**					
Non-smoker	57 (1.36%)	1.00	1.00	1.00	
Ex-smoker	42 (1.78%)	1.27 (0.80–2.03)	1.09 (0.67–1.77)	1.09 (0.67–1.76)	
Smoker	29 (1.64%)	1.26 (0.75–2.11)	1.33 (0.78–2.27)	1.34 (0.78–2.29)	
**Hypertension (*n*** **= 8490, failures = 105)**					
**PTSD**					
Non-case	92 (1.29%)	1.00	1.00	1.00	1.00
Case	11 (2.82%)	**2.22 (1.06–4.68)**	**2.56 (1.24–5.30)**	**2.23 (1.07–4.67)**	2.08 (0.94–4.62)
**CMD**					
Non-case	77 (1.29%)	1.00	1.00	1.00	1.00
Case	27 (1.75%)	1.37 (0.82–2.28)	1.50 (0.90–2.49)	1.39 (0.83–2.33)	1.35 (0.81–2.24)
**Alcohol misuse**					
Low risk drinking (0–7) ^d^	52 (1.60%)	1.00	1.00	1.00	
Hazardous drinking (8–15)	41 (1.22%)	0.78 (0.49–1.24)	1.04 (0.64–1.69)	1.00 (0.61–1.62)	
Harmful drinking/possible dependence (16+)	11 (1.31%)	0.86 (0.42–1.77)	1.54 (0.74–3.18)	1.39 (0.67–2.91)	
**Binge drinking at phase 2**	68 (1.50%)				
Monthly or less	36 (1.23%)	1.00	1.00	1.00	
Weekly or more		0.84 (0.53–1.33)	1.01 (0.63–1.62)	0.99 (0.62–1.58)	
**Smoking status at phase 2**					
Non-smoker	54 (1.42%)	1.00	1.00	1.00	
Ex-smoker	28 (1.50%)	1.04 (0.62–1.72)	0.81 (0.49–1.33)	0.77 (0.46–1.29)	
Smoker	22 (1.23%)	0.88 (0.50–1.55)	0.99 (0.56–1.75)	0.94 (0.53–1.67)	
**Prostate and GU disorders (*n*** **= 8469, failures = 90)**					
**PTSD**					
Non-case	80 (1.08%)	1.00	1.00	1.00	1.00
Case	9 (2.53%)	**2.42 (1.08–5.41)**	**2.50 (1.14–5.46)**	2.17 (0.97–4.85)	**2.47 (1.17–5.20)**
**CMD**					
Non-case	64 (1.00%)	1.00	1.00	1.00	1.00
Case	26 (1.75%)	**1.77 (1.04–3.00)**	**1.82 (1.09–3.05)**	**1.74 (1.05–2.89)**	**1.80 (1.08–3.01)**
**Alcohol misuse**					
Low risk drinking (0–7) ^d^	(*)	1.00	1.00	1.00	
Hazardous drinking (8–15)	(*)	1.14 (0.69–1.88)	1.22 (0.67–1.87)	1.11 (0.67–1.86)	
Harmful drinking/possible dependence (16+)	(*)	0.61 (0.25–1.49)	0.64 (0.25—1.66)	0.61 (0.24–1.59)	
**Binge drinking at phase 2**					
Monthly or less	62 (1.35%)	1.00	1.00	1.00	
Weekly or more	28 (0.84%)	0.63 (0.38–1.06)	0.63 (0.37–1.08)	0.63 (0.37–1.07)	
**Smoking status at phase 2**					
Non-smoker	39 (1.01%)	1.00	1.00	1.00	
Ex-smoker	25 (1.14%)	1.12 (0.61–2.02)	1.00 (0.54–1.83)	0.98 (0.54–1.80)	
Smoker	26 (1.47%)	1.51 (0.86–2.66)	1.35 (0.74–2.46)	1.37 (0.78–2.42)	

^a^Adjusted for age, gender, marital status, rank, serving status, engagement type, service branch, deployment to Iraq or Afghanistan and primary role in parent unit

^b^Additionally adjusted for family relationship adversity in childhood

^c^Additionally adjusted for alcohol misuse and smoking status at phase 2

^d^Category includes the never drinkers due to low cell sizes

(*) – Numbers and percentages are not reported when n is smaller than 8 due to Hospital Episode Statistics reporting guidelines

**Table 5:** Cox proportional hazards models examining the associations between change in CMD status and the NCD outcomes (*n* = 5794)

		**Gastrointestinal disorders (failures = 229)**	**Other joint disorders (failures = 196)**	**Arthritis/osteoarthritis (failures = 113)**	**Hypertension (failures = 92)**	**Prostate and other genitourinary disease (failures = 65)**
**CMD change**						
n (%)	Not a case stable	137 (59.45%)	132 (69.67%)	74 (69.09%)	55 (61.20%)	39 (59.89%)
	Case stable	29 (13.43%)	24 (12.19%)	14 (9.32%)	11 (7.98%)	12 (16.04%)
	Positive change	32 (12.96%)	20 (10.22%)	11 (11.66%)	14 (14.63%)	–
	Negative change	29 (14.15%)	15 (7.92%)	10 (9.93%)	11 (16.19%)	9 (17.18%)
**Unadjusted HR (95% CI)**	Not a case stable	1.00	1.00	1.00	1.00	1.00
	Case stable	**1.93 (1.28–2.93)**	1.44 (0.91–2.28)	1.55 (0.86–2.81)	1.65 (0.83–3.29)	**2.11 (1.08–4.12)**
	Positive change	**1.63 (1.09–2.43)**	1.00 (0.62–1.62)	0.92 (0.48–1.78)	1.82 (0.99–3.32)	–
	Negative change	**1.59 (1.05–2.42)**	0.75 (0.43–1.30)	1.03 (0.53–2.02)	1.53 (0.79–2.96)	1.59 (0.76–3.34)
**Fully adjusted HR (95% CI)**	Not a case stable	1.00	1.00	1.00	1.00	1.00
	Case stable	**1.98 (1.28–3.08)**	1.37 (0.85–2.22)	1.46 (0.76–2.79)	1.56 (0.77–3.16)	**2.02 (1.06–3.83)**
	Positive change	**1.65 (1.10–2.49)**	0.99 (0.60–1.64)	1.03 (0.52–2.01)	1.69 (0.89–3.21)	0.47 (0.13–1.65)
	Negative change	**1.77 (1.16–2.69)**	0.70 (0.40–1.23)	1.18 (0.59–2.37)	1.62 (0.85–3.10)	1.72 (0.81–3.68)

n (%) – unweighted frequencies and weighted percentages

Model 1 – unadjusted hazard ratios (95% CI)

Model 2 – hazard ratios (95% CI) adjusted for age, gender, marital status, rank, serving status, engagement type, service branch, deployment to Iraq or Afghanistan, primary role in parent unit and family relationship adversity in childhood, smoking status and alcohol use

#### Joint disorders

Smokers had 1.5 times the hazard of having an admission for a joint disorder compared to non-smokers, in the fully adjusted model. No other exposures were statistically significantly associated (Tables 4 and 5).

#### Arthritis/osteoarthritis

There were no associations with mental health, alcohol and smoking status with admissions for arthritis or osteoarthritis (Tables 4 & 5).

#### Hypertension

There was a statistically significant association between probable PTSD and hypertension (with 2 times the hazard of an admission), in some adjusted models but this was no longer significant in the fully adjusted model (after adjustment for alcohol misuse and smoking status) (Table 4).

#### Prostate or GU disorder

Individuals with probable PTSD and CMD had 2.5 and 1.8 times the hazard of having an admission for a prostate or GU disorder, in the fully adjusted model (Table 4). Personnel who met criteria for CMD across both phases had 2 times the hazard for an admission for prostate or other GU disorders, in the fully adjusted model (Table 5), compared to those who did not meet criteria for CMD at either phase.

#### Sensitivity analyses

The results of the sensitivity analyses in the matched only sample (*n* = 6336) examining associations between alcohol, smoking and mental health and the top 5 NCDs showed very similar results to the reported results in the full sample, with very small differences in the size of the hazard ratios and no difference in the statistical significance of the findings (See Supplementary table 2).

## Discussion

This is the first UK data linkage between a military cohort and secondary care records, and as far as we know is the first to link national healthcare records for England, Wales and Scotland together. The key finding is the prospective association between mental health and number of NCDs experienced which required a hospital admission, evidencing clear health inequalities compared to those with good mental health and the strong links between physical and mental health in this population. When examining specific NCDs, personnel with a mental disorder (probable PTSD or CMD) were found to be more likely to have admissions for GI disorders, hypertension and prostate & GU disorders. Smokers were also more likely to have admissions for GI disorders and joint disorders. There was no evidence that alcohol misuse was associated with admissions for NCDs, during the timeframe of this study.

Most of the serving and ex-serving personnel in this study did not have a hospital admission for an NCD, which might be expected given the average age. For those who did, GI disorders were the most common, followed by joint disorders which were more common than in the general population. The findings on GI disorders correspond to a UK general population data linkage study of the Hertfordshire cohort study to EHRs in older adults, which showed that digestive diseases were one of the most common conditions requiring an admission in males [53]. In addition to joint disorders, arthritis was also common in the older age groups, and this would be expected given the physical demands of the military occupation. UK research has shown that musculoskeletal disorders are the most common cause of medical discharge [54] and US data have shown a higher prevalence of arthritis in veterans compared to non-veterans [55].

One of the reasons why there is more US data on physical conditions that require treatment is because service related disability determines access to healthcare services, unlike in the UK. This VA data has found that hypertension was the most common condition requiring healthcare services in ex-serving personnel [17], whereas this was the fourth most common NCD in this UK sample. Coronary heart disease was also common in the US VA data but not in the current study, which is likely to be explained by the average younger age of the current sample. Other conditions such as diabetes and hearing problems, which have previously been shown to be common in ex-serving personnel [17], were not captured in the current data, most likely because they will require treatment in primary care, rather than leading to a hospital admission.

Military personnel with a mental disorder appear to have an increased risk of hospital admission for NCDs, specifically GI disorders, hypertension and prostate and GU disorders. Given that our previous work has established that at least a fifth of the UK military have a mental disorder [29], this is a sizeable proportion of this population who have a greater need of inpatient care for these physical conditions. The association with mental disorder and NCDs was stronger and more consistent across conditions for probable PTSD than CMD, indicating that this disorder could have a greater impact on physical health, potentially resulting from dysregulation of the autonomic nervous system due to the linked trauma exposure [56]. There are additional mechanisms through which PTSD could impact on physical health, identified in previous research, including 1) the impact on the inflammatory system [57], 2) specific effects on the cardiovascular system [58] and 3) through generally worsened health behaviours in those with a mental disorder, for example, higher levels of alcohol and smoking [59]. In the current study there was only evidence that alcohol and smoking may partially explain the association between PTSD and hypertension, suggesting this may be one of the mechanisms leading to increased blood pressure. Our findings generally support other studies in veterans, e.g. finding an association between PTSD and an increased number of physical health conditions in veterans [60], and PTSD was associated with reports of physical comorbidity in Australian Vietnam veterans [16]. Specifically, there is strong evidence for the association between PTSD and hypertension [61], but our findings on the risk of GI disorders and prostate and GU disorders have not previously been shown. There is a wealth of data on the association between physical and mental health in the general population; a recent study using Scottish hospital records showed that individuals with a mental disorder were twice as likely to have an emergency hospital admission (non-psychiatric) compared to those without [62]. It should be acknowledged that the associations identified in this study may also reflect increased rates of healthcare use in individuals with a mental disorder [63], and not only poorer health; but the fact that associations were not consistent across all physical health outcomes suggests that we have identified more than just an increased propensity for help seeking.

This study did not find an association between alcohol misuse and hospital admissions for NCDs in military personnel. This contradicts previous findings on the longer term health risks of alcohol [64], but may only reflect the fact that the alcohol harms have not yet reached the severity that requires an admission. There is no doubt in the literature of the physical harms for those drinking at a problematic level. The most recent data from NHS Digital for alcohol related hospital admissions suggests that the spike is for 45 to 55 and 55 to 64 year olds [65], which is older than the average age for this sample. The lack of association for hypertension was unexpected, given this is classified as being partially attributed to alcohol [66], but less severe cases of hypertension are likely to be treated in primary care and there is also evidence that blood pressure can reduce after alcohol use has been reduced [66].

Researchers in the alcohol field promote taking a life course approach to account for the fact that alcohol use may vary over time [67], but this won’t always capture the typical ups and downs of consumption that can occur between data collections. Particularly important for military personnel is the fact that they are more likely to have extended periods of abstention (for example, during deployments and advanced training operations) so their patterns of use may differ. Finally, being physically active may be protective against the health consequences of alcohol use e.g. for cancer [68], and given this population is likely to be physically active due to their occupation then this could explain these null effects. Further follow-up is required to see whether the association is stronger as these personnel age and reduce their levels of physical activity. In relation to other health behaviours, associations were found with smoking and the number of the NCDs requiring an admission, which is not a surprise, and the previous higher prevalence of smoking in military personnel [26, 27] has been proposed to explain the increased prevalence of coronary heart disease in this population [7].

### Strengths and weaknesses of the study

This study analysed data from a large and representative military cohort study and it involved the first UK linkage of military health data to secondary care (i.e. hospital) EHRs. The benefits of using this data linkage approach is that we were able to get objective health data on a large population, without intensive tracing and biomedical interviews. However, due to the limitations of these data only having reliable diagnosis codes in the admitted patient care data, outcomes in this study were largely around conditions which required surgery or an inpatient stay [38]. The EHRs for outpatient appointments may have provided the broadest picture of chronic health conditions, and the number of events would have been greater than for inpatient events providing increased statistical power to study rarer conditions, but unfortunately these data did not have adequate recording of ICD-10 diagnoses. Future research should also explore the possibility of linkage to primary care records which would cover more chronic, long term health conditions, that don’t require hospital care.

Health behaviours, including alcohol and smoking were self-reported in this study, which could result in a number of reporting biases (specifically under reporting of alcohol consumption given concerns about disclosing problems that may impact on career progression); however, alcohol misuse was assessed using a reliable and valid assessment tool [45] and other studies using the AUDIT have evidenced health risks for those drinking harmfully. The null findings for alcohol may relate to the lag time and age of the population and it is very possible that if we followed these individuals for another 10 to 20 years we may evidence an increase in alcohol related hospital admissions. Further limitations include the standardised comparisons to the general population, which couldn’t account for both gender and age together due to the restrictions of the data published by NHS Digital. Finally, the military cohort study had a modest response rate and we were only able to gain matched data on 73% of the military cohort. We took what was felt to be the conservative option in treating those who were not matched as not having a hospital episode, given that those who required NHS treatment were more likely to have an NHS number and therefore have been matched. Furthermore, the main findings did not differ regardless of whether the matched or full sample was included in the analysis.

### Clinical implications

These results provide strong evidence that within an AF population, those with a mental health problem have worse physical health. This highlights the priority to provide good mental healthcare and quick access to mental health services for serving personnel and veterans, to reduce the impact on their longer term physical health. The increased risk of hypertension for those with probable PTSD appeared to be partly explained by higher levels of smoking and alcohol use (given that the association attenuated after adjustment for these variables), suggesting that more effective health promotion and behaviour change interventions for those with a mental health problem may be required. Even though the prevalence of smoking is typically lower in the current UK military population compared to civilians, targeted interventions to encourage cessation in those who do smoke will have positive health consequences.

## Conclusions

Military personnel with a mental health problem are more likely to have an inpatient hospital admission for NCDs compared to those without, specifically for gastrointestinal disorders, hypertension and prostrate and GU disorders. For hypertension, this increased risk appears to be partially driven by poorer health behaviours. This study has shown that it is feasible to link a military cohort to admitted patient care records from England, Wales and Scotland and to identify which conditions most commonly require inpatient care in this population.

## Supplementary information

**Additional file 1: Supplementary Table 1.** Comparing personnel who were matched and unmatched to English, Scottish or Welsh electronic healthcare records. **Supplementary Table 2.** Cox proportional hazards models examining the associations with mental health, alcohol misuse, smoking for the top 5 NCDs (restricted to the matched sample).

**Additional file 2. Supplementary file 1.** Developing a framework of non-communicable physical diseases.

**Additional file 3. Supplementary figure 1.** Figure showing data linkage of the KCMHR cohort data to the electronic healthcare records.

## Data Availability

The data that support the findings of this study are available from NHS Digital for England, NHS Information Services for Wales and Information Division Services for Scotland for the electronic healthcare records and from King’s Centre for Military Health Research for the cohort data, but restrictions apply to the availability of these data, which were used under license for the current study, and so are not publicly available. Data are however available from the authors upon reasonable request and with permission of NHS Digital, NHS Information Services for Wales, Information Division Services for Scotland and King’s Centre for Military Health Research.

## Abbreviations

NCD: Non-communicable disease
PTSD: Post-traumatic stress disorder
CMD: Common mental disorder
EHR: Electronic healthcare records
NHS: National health services
KCMHR: King’s centre for military health research
HR: Hazard ratio
CI: Confidence intervals
