# Supplementary material for: Hospital admissions for non-communicable disease in the UK military and associations with alcohol use and mental health: a data linkage study
Source: BMC Public Health. 2020 Sep 10;20:1236. doi: 10.1186/s12889-020-09300-5 (PMC7488237; doi:10.1186/s12889-020-09300-5)
Supplement: Supplementary file 1 — Additional file 1: Supplementary Table 1. Comparing personnel who were matched and unmatched to English, Scottish or Welsh electronic healthcare records. Supplementary Table 2. Cox proportional hazards models examining the associations with mental health, alcohol misuse, smoking for the top 5 NCDs (restricted to the matched sample). [file 12889_2020_9300_MOESM1_ESM.docx]

**Supplementary Table 1: Comparing personnel who were matched and unmatched to English, Scottish or Welsh electronic healthcare records**

|  | **Matched sample (n=6336)** | | **Unmatched sample (n=2266)** | | **Comparison between the matched and unmatched sample** |
| --- | --- | --- | --- | --- | --- |
|  | n | **Weighted Col %** | n | **Weighted Col %** | **Chi-square (p)** |
| **Age in years at phase 2 (mean, S.D.)** | 35.57 (8.99) | | 37.26 (9.65) | | p=0.000 |
| **Sex at sampling** |  |  |  |  |  |
| Male | 5615 | 90.40% | 2046 | 90.77% | 0.20 (p=0.65) |
| Female | 721 | 9.60% | 220 | 9.23% |  |
| **Relationship status at phase 2** |  |  |  |  |  |
| Married/in a relationship | 4828 | 78.38% | 1774 | 80.95% | 2.63 (p=0.07) |
| Single | 1051 | 14.01% | 352 | 12.67% |  |
| Separated/Divorced/  Widowed | 438 | 7.61% | 132 | 6.38% |  |
| **Rank at phase 2** |  |  |  |  |  |
| Officers | 1464 | 20.14% | 466 | 19.74% | 0.13 (p=0.72) |
| NCOs/Other Ranks | 4,872 | 79.86% | 1800 | 80.26% |  |
| **Left service marker (at phase 2)** |  |  |  |  |  |
| Serving | 4797 | 71.71% | 1674 | 70.36% | 1.04 (p=0.31) |
| Discharged | 1526 | 28.29% | 591 | 29.64% |  |
| **Regular/Reserve status** |  |  |  |  |  |
| Regular | 5169 | 88.74% | 1890 | 88.69% | 0.00 (p=0.95) |
| Reserve | 1167 | 11.26% | 376 | 11.31% |  |
| **Service** |  |  |  |  |  |
| Naval Services | 841 | 14.45% | 476 | 21.64% | **130.75 (p=0.00)** |
| Army | 4505 | 70.06% | 1123 | 48.19% |  |
| RAF | 990 | 15.50% | 667 | 30.16% |  |
| **Deployed before phase 2** |  |  |  |  |  |
| No deployment | 2014 | 42.30% | 795 | 46.99% | **4.25 (p=0.01)** |
| Iraq only | 2743 | 37.93% | 953 | 35.18% |  |
| Afghanistan only | 685 | 8.93% | 232 | 8.52% |  |
| Both Iraq and Afghanistan | 894 | 10.84% | 236 | 9.31% |  |
| **Role in parent unit** |  |  |  |  |  |
| Combat | 1580 | 25.54% | 455 | 19.90% | **10.68 (p=0.00)** |
| Combat support | 729 | 11.17% | 282 | 12.06% |  |
| Combat service support | 3959 | 63.29% | 1507 | 68.04% |  |
| **Family relationship adversities in childhood** |  |  |  |  |  |
| 0/1 | 4020 | 63.81% | 1459 | 65.31% | 1.31 (p=0.27) |
| 2/3 | 1260 | 19.89% | 460 | 20.09% |  |
| 4+ | 985 | 16.30% | 326 | 14.60% |  |
| **Self-reported health** |  |  |  |  |  |
| Poor/Fair | 769 | 13.37% | 231 | 11.00% | **5.78 (p=0.02)** |
| Good/Excellent | 5537 | 86.63% | 2030 | 89.00% |  |
| **Alcohol misuse** |  |  |  |  |  |
| Current never drinkers | 103 | 1.41% | 68 | 3.34% | **14.43 (p=0.00)** |
| Low risk drinking (0-7) | 2499 | 40.40% | 1000 | 44.79% |  |
| Hazardous drinking (8-15) | 2766 | 44.22% | 923 | 41.51% |  |
| Harmful drinking/possible dependence (16+) | 891 | 13.96% | 247 | 10.36% |  |
| **GHQ case (3/4)** |  |  |  |  |  |
| No | 4996 | 79.33% | 1838 | 81.89% | **4.88 (p=0.03)** |
| Yes | 1282 | 20.67% | 404 | 18.11% |  |
| **PCL case (49/50)** |  |  |  |  |  |
| No | 6027 | 95.72% | 2172 | 96.35% | 1.17 (p=0.28) |
| Yes | 261 | 4.28% | 76 | 3.65% |  |
| **NHS number recorded** |  |  |  |  |  |
| No | 611 | 9.77% | 1114 | 46.24% | **1018.04 (p=0.00)** |
| Yes | 5725 | 90.23% | 1152 | 53.76% |  |

**Supplementary Table 2: Cox proportional hazards models examining the associations with mental health, alcohol misuse, smoking for the top 5 NCDs (restricted to the matched sample)**

|  | **Unadjusted Hazard Ratio (HR) (95% CI)** | **Adjusted HR (95% CI)^1^** | **Adjusted HR (95% CI)^2^** | **Adjusted HR (95% CI)^3^** |
| --- | --- | --- | --- | --- |
| **Gastrointestinal disorders (n= 6111, failures=303)** | | | | |
| **PTSD** |  |  |  |  |
| Non-case | 1.00 | 1.00 | 1.00 | 1.00 |
| Case | **2.23 (1.34-3.71)** | **2.18 (1.29-3.70)** | **2.17 (1.26-3.74)** | **2.31 (1.33-3.98)** |
| **CMD** |  |  |  |  |
| Non-case | 1.00 | 1.00 | 1.00 | 1.00 |
| Case | **1.42 (1.03-1.94)** | **1.43 (1.05-1.96)** | **1.44 (1.05-1.97)** | **1.51 (1.09-2.09)** |
| **Alcohol misuse** |  |  |  |  |
| Low risk drinking (0-7)^±^ | 1.00 | 1.00 | 1.00 |  |
| Hazardous drinking (8-15) | 0.87 (0.66-1.16) | 1.02 (0.75-1.38) | 1.01 (0.74-1.38) |  |
| Harmful drinking/possible dependence (16+) | 0.79 (0.50-1.26) | 1.06 (0.65-1.71) | 1.04 (0.63-1.72) |  |
| **Binge drinking at phase 2** |  |  |  |  |
| Monthly or less | 1.00 | 1.00 | 1.00 |  |
| Weekly or more | **0.67 (0.50-0.89)** | 0.75 (0.56-1.02) | 0.75 (0.55-1.02) |  |
| **Smoking status at phase 2** |  |  |  |  |
| Non-smoker | 1.00 | 1.00 | 1.00 |  |
| Ex-smoker | 1.31 (0.95-1.80) | 1.18 (0.85-1.64) | 1.17 (0.84-1.63) |  |
| Smoker | **1.43 (1.03-1.99)** | **1.59 (1.15-2.21)** | **1.59 (1.14-2.21)** |  |
| **Joint disorders (n= 6113, failures=291)** | | | | |
| **PTSD** |  |  |  |  |
| Non-case | 1.00 | 1.00 | 1.00 | 1.00 |
| Case | 1.19 (0.65-2.18) | 1.22 (0.66-2.26) | 1.18 (0.64-2.18) | 1.06 (0.57-1.98) |
| **CMD** |  |  |  |  |
| Non-case | 1.00 | 1.00 | 1.00 | 1.00 |
| Case | 1.04 (0.74-1.46) | 1.05 (0.74-1.47) | 1.03 (0.73-1.45) | 0.99 (0.70-1.42) |
| **Alcohol misuse** |  |  |  |  |
| Low risk drinking (0-7) ^±^ | 1.00 | 1.00 | 1.00 |  |
| Hazardous drinking (8-15) | 0.99 (0.74-1.32) | 0.94 (0.69-1.28) | 0.94 (0.69-1.27) |  |
| Harmful drinking/possible dependence (16+) | 1.32 (0.88-1.99) | 1.24 (0.80-1.93) | 1.24 (0.79-1.93) |  |
| **Binge drinking at phase 2** |  |  |  |  |
| Monthly or less | 1.00 | 1.00 | 1.00 |  |
| Weekly or more | 1.22 (0.93-1.61) | 1.19 (0.89-1.58) | 1.19 (0.89-1.59) |  |
| **Smoking status at phase 2** |  |  |  |  |
| Non-smoker | 1.00 | 1.00 | 1.00 |  |
| Ex-smoker | 1.23 (0.88-1.72) | 1.23 (0.88-1.73) | 1.23 (0.88-1.72) |  |
| Smoker | **1.50 (1.09-2.08)** | **1.47 (1.05-2.07)** | **1.46 (1.04-2.06)** |  |
| **Arthritis/osteoarthritis (n= 6236, failures=131)** | | | | |
| **PTSD** |  |  |  |  |
| Non-case | 1.00 | 1.00 | 1.00 | 1.00 |
| Case | 1.48 (0.65-3.37) | 1.80 (0.78-4.12) | 1.85 (0.81-4.20) | 1.74 (0.70-4.32) |
| **CMD** |  |  |  |  |
| Non-case | 1.00 | 1.00 | 1.00 | 1.00 |
| Case | 1.06 (0.65-1.70) | 1.15 (0.71-1.85) | 1.17 (0.72-1.88) | 1.06 (0.64-1.78) |
| **Alcohol misuse** |  |  |  |  |
| Low risk drinking (0-7) ^±^ | 1.00 | 1.00 | 1.00 |  |
| Hazardous drinking (8-15) | 0.74 (0.48-1.13) | 0.91 (0.58-1.42) | 0.91 (0.58-1.44) |  |
| Harmful drinking/possible dependence (16+) | 0.63 (0.30-1.34) | 0.92 (0.42-2.04) | 0.96 (0.43-2.15) |  |
| **Binge drinking at phase 2** |  |  |  |  |
| Monthly or less | 1.00 | 1.00 | 1.00 |  |
| Weekly or more | 0.83 (0.54-1.28) | 0.98 (0.62-1.53) | 0.99 (0.63-1.56) |  |
| **Smoking status at phase 2** |  |  |  |  |
| Non-smoker | 1.00 | 1.00 | 1.00 |  |
| Ex-smoker | 1.26 (0.79-2.00) | 1.08 (0.67-1.75) | 1.08 (0.67-1.75) |  |
| Smoker | 1.16 (0.69-1.95) | 1.28 (0.75-2.20) | 1.29 (0.75-2.23) |  |
| **Hypertension (n= 6242, failures=105)** | | | | |
| **PTSD** |  |  |  |  |
| Non-case | 1.00 | 1.00 | 1.00 | 1.00 |
| Case | **2.13 (1.01-4.49)** | **2.56 (1.24-5.26)** | **2.23 (1.07-4.64)** | 2.03 (0.87-4.76) |
| **CMD** |  |  |  |  |
| Non-case | 1.00 | 1.00 | 1.00 | 1.00 |
| Case | 1.31 (0.79-2.19) | 1.43 (0.86-2.39) | 1.34 (0.79-2.25) | 1.28 (0.77-2.15) |
| **Alcohol misuse** |  |  |  |  |
| Low risk drinking (0-7) ^±^ | 1.00 | 1.00 | 1.00 |  |
| Hazardous drinking (8-15) | 0.74 (0.46-1.17) | 1.04 (0.63-1.71) | 0.99 (0.61-1.63) |  |
| Harmful drinking/possible dependence (16+) | 0.77 (0.37-1.59) | 1.50 (0.73-3.10) | 1.36 (0.65-2.83) |  |
| **Binge drinking at phase 2** |  |  |  |  |
| Monthly or less | 1.00 | 1.00 | 1.00 |  |
| Weekly or more | 0.80 (0.51-1.28) | 1.02 (0.63-1.64) | 0.98 (0.61-1.57) |  |
| **Smoking status at phase 2** |  |  |  |  |
| Non-smoker | 1.00 | 1.00 | 1.00 |  |
| Ex-smoker | 1.03 (0.62-1.71) | 0.85 (0.51-1.40) | 0.81 (0.48-1.35) |  |
| Smoker | 0.81 (0.46-1.42) | 0.98 (0.55-1.74) | 0.93 (0.52-1.66) |  |
| **Prostate and GU disorders (n= 6221, failures=90)** | | | | |
| **PTSD** |  |  |  |  |
| Non-case | 1.00 | 1.00 | 1.00 | 1.00 |
| Case | **2.33 (1.04-5.22)** | **2.48 (1.14-5.43)** | 2.19 (0.98-4.92) | **2.43 (1.15-5.13)** |
| **CMD** |  |  |  |  |
| Non-case | 1.00 | 1.00 | 1.00 | 1.00 |
| Case | **1.70 (1.00-2.88)** | **1.75 (1.05-2.94)** | **1.69 (1.02-2.81)** | **1.75 (1.05-2.93)** |
| **Alcohol misuse** |  |  |  |  |
| Low risk drinking (0-7) ^±^ | 1.00 | 1.00 | 1.00 |  |
| Hazardous drinking (8-15) | 1.08 (0.66-1.79) | 1.09 (0.65-1.82) | 1.08 (0.65-1.81) |  |
| Harmful drinking/possible dependence (16+) | 0.55 (0.22-1.34) | 0.61 (0.23—1.59) | 0.58 (0.22-1.52) |  |
| **Binge drinking at phase 2** |  |  |  |  |
| Monthly or less | 1.00 | 1.00 | 1.00 |  |
| Weekly or more | 0.61 (0.36-1.02) | 0.62 (0.36-1.06) | 0.62 (0.36-1.06) |  |
| **Smoking status at phase 2** |  |  |  |  |
| Non-smoker | 1.00 | 1.00 | 1.00 |  |
| Ex-smoker | 1.10 (0.61-2.01) | 1.00 (0.54-1.82) | 0.98 (0.54-1.80) |  |
| Smoker | 1.40 (0.79-2.46) | 1.35 (0.76-2.43) | 1.33 (0.75-2.36) |  |
|  | | | | |
| ^1^Adjusted for age, gender, marital status, rank, serving status, engagement type, service branch, deployment to Iraq or Afghanistan and primary role in parent unit  ^2^Additionally adjusted for family relationship adversity in childhood  ^3^Additionally adjusted for alcohol misuse and smoking status at phase 2  ^±^ Category includes the never drinkers due to low cell sizes | | | | |
