# Supplementary material for: Hospital admissions for non-communicable disease in the UK military and associations with alcohol use and mental health: a data linkage study
Source: BMC Public Health. 2020 Sep 10;20:1236. doi: 10.1186/s12889-020-09300-5 (PMC7488237; doi:10.1186/s12889-020-09300-5)
Supplement: Supplementary file 2 — Additional file 2. Supplementary file 1. Developing a framework of non-communicable physical diseases. [file 12889_2020_9300_MOESM2_ESM.docx]

**Additional file 1**

***Developing a framework of non-communicable physical diseases – i) overview of the search, ii) flow diagram and iii) finalised framework of non-communicable disease***

***i)Outline of search:***

Database search: A summary of the search process is presented below. Three databases were searched on 16 November 2015 via the Ovid research tool: PsycINFO (1806–present), MedLine (1946–present) and Embase (1980–present). Each database was searched using free text keywords. The keywords “multimorbidity” and “multi-morbidity” were included as search criterion in all fields. The chronic conditions aspect of the search included the keywords “chronic disease” OR “chronic illness”. After removing duplicates, 613 references went through to abstract review. Abstract review: At the abstract review stage, studies that were not directly relevant to the present study were excluded, reducing the number of studies from 613 to 138. Common themes of studies that were excluded were the following: (1) studies where the full text was not available in English; (2) studies limited to a specific illness population; (3) studies in which the focus of the paper is the management of multimorbidity in the long term (4) studies of multimorbidity with mental health conditions (5) studies of multimorbidity with infectious diseases. Inclusion and exclusion criteria: Studies were included according to the following inclusion criteria: (1) studies which investigate the multimorbidity of chronic conditions in a representative sample (defined as the existence of several chronic health disorders in one individual); (2) studies that generated a list of chronic conditions based on prevalence data provided by national health services and primary care practices. Studies were excluded if: (1) they did not provide any justification for the selection of chronic conditions included in the study; (2) the definition of “chronic” or “multimorbid” was not stated or was inadequate (3) they only examined 7 conditions or fewer (based on Fortin et al. (2012)’s recommendation); (4) no list had been provided. For example, studies which investigated classifying disorders or only reported domains (with no specific conditions within these domains mentioned); (5) they referred to lists generated in previous studies. After applying these criteria, 34 studies were identified as being relevant to the present study and a further 17 studies were identified from references thus providing a total of 51 lists of chronic conditions. Of these 51 lists, 7 used ICD-10 or ICD-9 codes. ICD-10 codes and groupings: A framework of non-communicable physical diseases was developed by collating the 51 lists and ordering the conditions from most to least common. Conditions were included if they appeared in more than two lists to avoid rare or specific conditions. Decisions on how certain conditions would be grouped together and the ICD-10 codes assigned to these groups were informed by the literature search, ICD-10 codes used in previous studies, health information websites such as *NHS Choices* and the coding structure in ICD-10. Condition groupings were assessed for face validity by four clinicians within the Department of Psychological Medicine, King’s College London. The final framework included 28 non-communicable physical conditions which fall under ten disease categories.

**ii) Flow diagram of search process:**


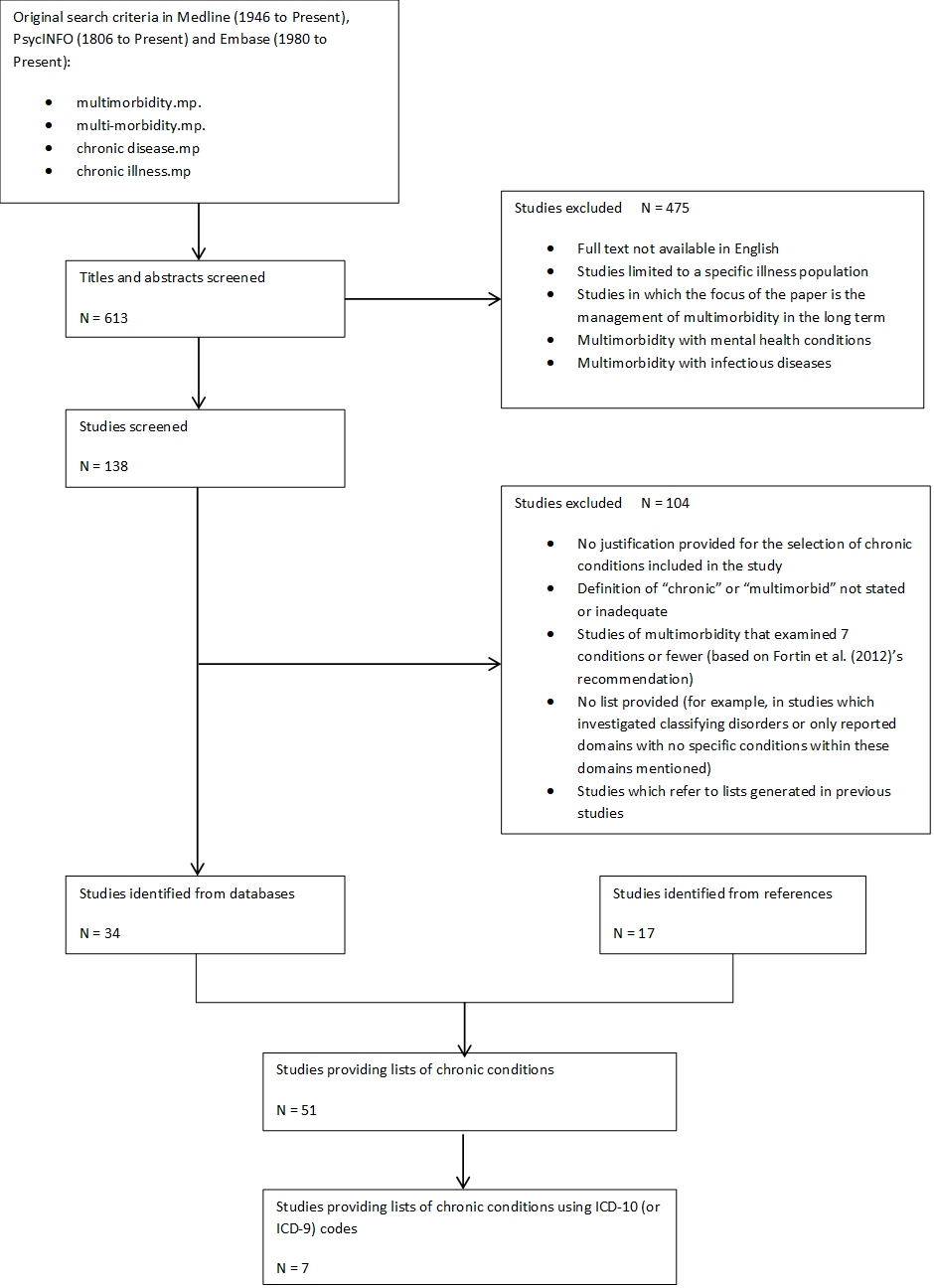


**iii) Non-communicable disease framework – 28 conditions under 10 disease categories**

|  | **Condition** | **ICD-10 codes** |
| --- | --- | --- |
|  | **Cancer** |  |
| **1** | Cancer and tumours | C00-C97, D00-D09, D37-D47 |
|  | **Haematological** |  |
| **2** | Anaemia | D50-D53, D55-D61, D63, D64 |
|  | **Endocrine** |  |
| **3** | Thyroid disorders | E01-E07 |
| **4** | Diabetes (Type I) | E10 |
| **5** | Diabetes (Type II) | E11-E14 |
|  | **Obesity/hyperlipidaemia** |  |
| **6** | Obesity | E66 |
| **7** | Hyperlipidaemia | E78 |
|  | **Neurological** |  |
| **8** | Dementia | F00-F03 |
| **9** | Neurodegenerative diseases^1^ | G10, G11, G12, G20 |
| **10** | Epilepsy | G40 |
| **11** | Migraine/headache | G43, G44, R51 |
| **12** | Hearing loss or vision impairment | H17, H18, H25-H28, H31, H33-H36, H40, H43, H47, H54, H90, H91 |
|  | **Cardiovascular** |  |
| **13** | Coronary heart disease (CHD)^2^ | I05-I09, I20-I25 |
| **14** | Hypertension | I10-I15 |
| **15** | Cardiomyopathy | I42- I43 |
| **16** | Cardiac conduction abnormalities | I44-I45, I47-I49 |
| **17** | Heart failure | I50 |
| **18** | Cerebrovascular disease^3^ | I60-I69 |
| **19** | Peripheral vascular disease | I70, I73-I79 |
|  | **Respiratory** |  |
| **20** | Asthma/COPD | J40-J47 |
|  | **Gastrointestinal** |  |
| **21** | Gastrointestinal disorders^4^ | K20-K31, K50-K52, K55-K64 |
| **22** | Liver disease | K70-K77 |
|  | **Musculoskeletal** |  |
| **23** | Arthritis/osteoarthritis | M05-M06, M15-M19 |
| **24** | Other joint disorders | M20-M25 |
| **25** | Back and neck pain^5^ | M54 |
| **26** | Osteoporosis^6^ | M80-M85 |
|  | **Genitourinary** |  |
| **27** | Chronic kidney disease/renal failure | N17-N19 |
| **28** | Prostate and other genitourinary disease^7^ | N40-N45, N47-N51 |

1 = Neurodegenerative diseases including Huntington disease, hereditary ataxia, spinal muscular atrophy and related syndromes and Parkinson disease

2 = Coronary heart disease including ischemic heart disease, chronic rheumatic heart disease, coronary artery disease, myocardial infarction and angina (excluding heart valve disease)

3 = Cerebrovascular disease including stroke

4 = Gastrointestinal disorders including gastroesophageal reflux disease (GERD), functional dyspepsia, inflammatory bowel disease (including Crohn’s disease and ulcerative colitis), diverticular disease of the intestine, peptic ulcer, irritable bowel syndrome and other functional intestinal disorders (e.g. constipation)

5 = Back and neck pain including panniculitis affecting regions of neck and back, radiculopathy, cervicalgia, sciatica, lumbago with sciatica, low back pain and pain in thoracic spine

6 = Osteoporosis and pathological fracture

7 = Prostate and other genitourinary disease including hyperplasia of prostate, other disorders of prostate, hydrocele and spermatocele, torsion of testis, orchitis and epididymitis, redundant prepuce, phimosis and paraphimosis, other disorders of penis, inflammatory disorders of male genital organs and other disorders of male genital organs.
