# Supplementary figures and images for: Hospital admissions for non-communicable disease in the UK military and associations with alcohol use and mental health: a data linkage study
Source: BMC Public Health. 2020 Sep 10;20:1236. doi: 10.1186/s12889-020-09300-5 (PMC7488237; doi:10.1186/s12889-020-09300-5)

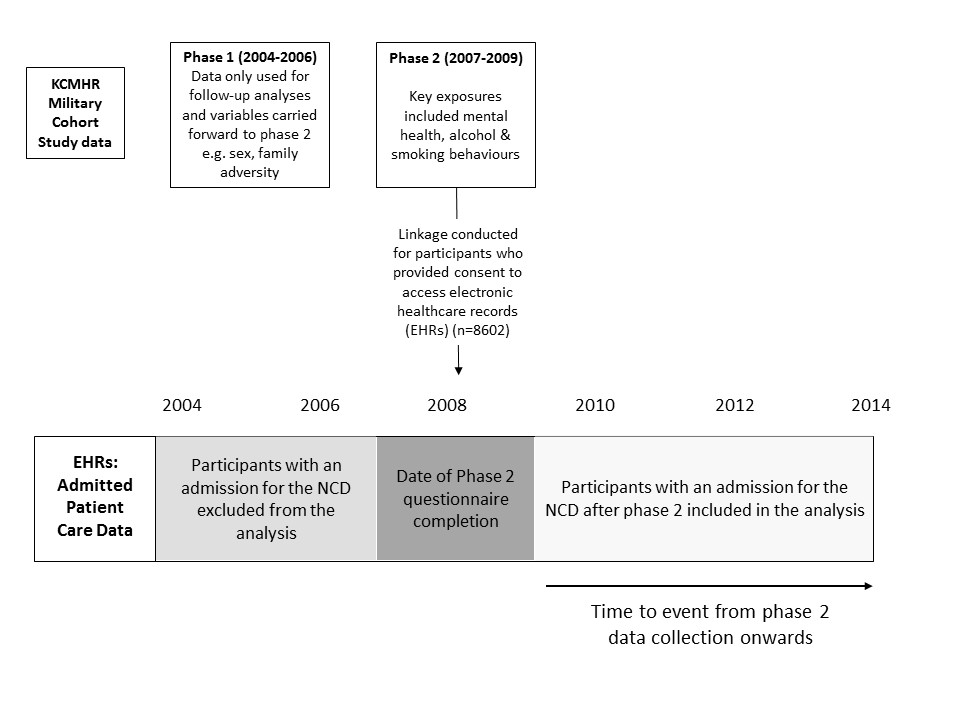

Supplement: Supplementary file 3 — Additional file 3. Supplementary figure 1. Figure showing data linkage of the KCMHR cohort data to the electronic healthcare records. [file 12889_2020_9300_MOESM3_ESM.jpg]
